# The Military Surgeon

**Published:** 1909-04

**Authors:** 


### BUFFALO MEDICAL JOURNAL.

**A Monthly Review of Medicine and Surgery.**

*EDITOR:*

WILLIAM WARREN POTTER, M. D.

All communications, whether of a literary or business nature, books for review and exchanges, should be addressed to the editor, 238 Delaware Avenue, Buffalo, N. Y.

Vol. LXIV. APRIL, 1909. No. 9

### The Military Surgeon

WITH the current issue of *The Military Surgeon*, Major James Evelyn Pilcher, its founder, and secretary of the Association of Military Surgeons of the United States, retires from editorial management and leaves to his successor, Major Charles Lynch, of the medical department of the army, a journal of military medicine which has not its equal in the world. Major Pilcher for years endeavored to secure the coöperation of the army authorities in the establishment of a journal devoted to military medicine, but met with little or no encouragement. There was that constant beaurocratic offishness, whenever the subject was mentioned, which was quite as lethal in its effect as would have been official condemnation. However, with the organisation of the Association, the doors were opened and the journal was established by Dr. Pilcher in 1901 and conducted by him up to the present time, in connection with his work as secretary of the Association.

Step by step he upbuilded the journal; its influence was widened; its pages were contributed to by the foremost authorities on military medicine in the world; no country boasting of an army and an army medical department was unrepresented in its volumes. All this time, Dr. Pilcher who had been retired from the medical department of the army because of disability incurred in the service, was working against the distressing odds of ill-health. And there is no doubt that his period of activity has been shortened by the devoted work he gave to the conduct of the journal which he has brought to its present position of eminence and power.

An idea of the terrible difficulties under which Major Pilcher has been laboring may be gathered when it is known that for two years he has not been able to read a line of manuscript submitted for publication nor a word of the printed journal because of chronic retinitis, a complication of the disability for which he was retired. This exhibition of enforced dependence, unbearable to a man of Dr. Pilcher's activities, together with an enormous increase in the secretarial work made it imperative that he take that comfort which only absolute rest would give him. His request for relief was made to the executive council of the Association and his resignation was accepted with the adoption of these resolutions prepared by the three senior ex-presidents of the Association of Military Surgeons:

WHEREAS, owing to increasing physical infirmity, due to long and faithful service, Major James Evelyn Pilcher has found it necessary to tender his resignation as secretary of the Association and editor of the journal; and

WHEREAS, in the discharge of his duties, this officer has earned a high reputation for editorial ability and scholarship, also establishing for the Journal an authoritative position in medico-military literature; and

WHEREAS, he has, by his untiring industry in behalf of the Association, greatly extended its usefulness and welfare, be it therefore,

*Resolved,* by the Executive Council, That in the resignation of Major Pilcher the Association loses the services of a valuable officer.

*Resolved,* That wishing to give formal expression to our esteem and the regret we experience, not only at his resignation, but the condition rendering it necessary, this action be communicated to Major Pilcher, and that it be published in the journal of the association.

GEO. M. STERNBERG, U.S.A.

WALTER WYMAN, U.S.P.H.& M.H.S.

JOHN C. WISE, U.S.N.

This formal expression of regret, sincere as it is, coming as it does in the shape of resolutions, but faintly speaks the deep sense of loss which the Association will experience in the retirement of Major Pilcher. He has devoted to the work of editing the journal years of his life, none of which was free from illness and suffering; and that he succeeded in doing the great work which he has accomplished under such adverse circumstances speaks loud for his self-sacrificing devotion and his courage. There are few men who could, or would, have withstood the strain. There are still fewer who could, as Major Pilcher has on several occasions, stand before a gathering of strangers at the public sessions when the foreign delegates were decorated with the insignia of the Association, and in spite of serious illness, in the presence of pain, make presentation speeches which scintillated with wit, shone with historic reference and sparkled with rhetoric brilliancy.

Such a man is Pilcher and in his retirement he takes with him the heartiest wishes of his friends and associates for a speedy improvement in health.

The new editor of *The Military Surgeon*, Major Charles Lynch of the army, is well known in Buffalo, having served as surgeon at Fort Porter a few years ago. There could have been no better selection for secretary of the Association and editor of *The Military Surgeon* than Major Lynch. A man of high literary attainments and wide experience in military medicine, he will bring to the conduct of the journal all the elements which are necessary for the continued up-building of the finest medico-military journal in the world.

Major Lynch was the Army Medical Department representative with the Japanese army during the Russo-Japanese war; he won the Enno Sander medal for 1907, and recently has been active in the organisation of the medical reserve corps of the medical department of the Army.

We take leave of Pilcher with sincere regret; we welcome Lynch with warm friendship and affectionate regard.

The lines of *The Military Surgeon* have fallen in strong hands. N. W. W.

